# Compositional changes in bee and wasp communities along Neotropical mountain altitudinal gradient

**DOI:** 10.1371/journal.pone.0182054

**Published:** 2017-07-26

**Authors:** Lucas Neves Perillo, Frederico de Siqueira Neves, Yasmine Antonini, Rogério Parentoni Martins

**Affiliations:** 1 Departamento de Biologia Geral, Instituto de Ciências Biológicas, Universidade Federal de Minas Gerais, Belo Horizonte, MG, Brazil; 2 Departamento de Biodiversidade Evolução e Meio Ambiente, Universidade Federal de Ouro Preto, Ouro Preto, MG, Brazil; 3 Programa de Pós-Graduação em Ecologia e Recursos Naturais, Universidade Federal do Ceará, Fortaleza, CE, Brazil; Natural Resources Canada, CANADA

## Abstract

Climate conditions tend to differ along an altitudinal gradient, resulting in some species groups’ patterns of lower species richness with increasing altitude. While this pattern is well understood for tropical mountains, studies investigating possible determinants of variation in beta-diversity at its different altitudes are scarce. We sampled bee and wasp communities (Hymenoptera: Aculeata) along an altitudinal gradient (1,000–2,000 m.a.s.l.) in a tropical mountainous region of Brazil. Trap nests and Moericke traps were established at six sampling points, with 200 m difference in altitude between each point. We obtained average climate data (1970–2000) from Worldclim v2 for altitudes at each sampling site. Nest traps captured 17 bee and wasp species from six families, and Moericke traps captured 124 morphospecies from 13 families. We found a negative correlation between altitude and species richness and abundance. Temperature, precipitation, water vapor pressure, and wind speed influenced species richness and abundance, and were correlated with altitude. β-diversity was primarily determined by species turnover as opposed to nestedness, and Aculeate community similarity was higher for more similar altitudinal ranges. Moericke traps seem to be more efficient for altitudinal surveys compared to nest traps. We found high occurrence of singleton and doubleton species at all altitudes, highlighting the need for long-term studies to efficiently assess hymenopteran diversity in these environments.

## Introduction

Beta-diversity (β) is a property of complex biological communities [1,2], and is inherently connected to large-scale measurements of species richness (i.e., gamma-diversity) through the species–area relationship found for nearly all organisms investigated thus far [3,4]. However, less attention has been paid to patterns of β-diversity [5–7], and the relative importance of environmental and geographic variables for beta diversity remains controversial. Although the importance of β-diversity to gamma-diversity has been investigated for several taxa and environments [5,8,9], it is much less understood than, for example, gradients in species richness or within-habitat diversity (i.e., alpha-diversity). Studies of tropical invertebrate β-diversity mostly emphasize habitat differences that influence species richness [10], and often fail to detect spatial effects at smaller spatial scales [11,12].

Beta-diversity can be decomposed into turnover (species replacement between sites) and nestedness (species loss or gain between sites) components [13]. Distinction between components is particularly important in threatened environments such as mountainous regions [14], where evaluation of the mechanisms involved in each may improve our descriptions of species spatial distributions [15]. Beta-diversity varies by altitude, and should be highest in heterogeneous habitats (e.g., tropical mountains) due to higher numbers of coexisting habitat specialists [16]. Studies of diversity patterns in tropical mountains mostly show a decline in plant and invertebrate species richness with increasing altitude [14,17–22]. This pattern may arise as a response to changes in weather conditions, such as wind speed, light intensity, humidity, and specially temperature [22]. Other factors such as reduction in habitat area, resource diversity, and primary productivity may also result in lower diversity at higher elevations [23–28]. Species richness does not always decline linearly with increasing altitude, as some studies indicate a hump-shaped pattern with peaks in diversity at intermediate altitudes (e.g., as caused by the mid-domain effect) (see [29] for more examples). Species richness may also increase with altitude, which has been documented for free-feeding herbivores in mesic habitats in Brazil [30,31] and parasitoid wasps (Hymenoptera: Ichneumonidae) in the Costa Rican low mountain ranges [32].

Bees and wasps are excellent model organisms for investigating how environmental variation along an altitudinal gradient influences insect beta-diversity. First, climatic variables and weather conditions are particularly important for insects [33,34], especially because thermoregulation capability is determined by ambient temperature [35,36]. Furthermore, bee and wasp diversity is strongly correlated with the availability of food and nesting resources [33,37,38], which are usually more scarce at high elevations (i.e., mountaintops) [38–41]. Bee and wasp diversity thus should decrease with increasing altitude (e.g., [42]). There are a few studies on insect distributions in old mountaintop ecosystems in Brazil, called ‘*campos rupestres*’ and ‘*campos de altitude*’ [14,31,43]. Among these studies the Aculeata clade is particularly well described (Hymenoptera: Apocrita) (see [44,45] for phylogeny) (e.g., [20,46–51]).

In this study we investigated bee and wasp species composition and richness along a tropical mountain altitudinal gradient. We specifically tested: 1. whether species richness decreases with increasing altitude along the gradient; 2. whether species composition changes with increasing altitude; and 3. whether the observed β-diversity patterns are primarily due to species turnover or nestedness.

## Materials and methods

### Study sites

The study was developed in *Reserva Particular do Patrimônio Natural Santuário do Caraça* (hereafter the “Caraça Mountains”) in Minas Gerais, Brazil (20°05'54" S, 43°29'17" W). The study area lies at the southern limit of the Espinhaço mountain range, which is the largest and one of the most important mountain formations (i.e., in terms of biodiversity) in Brazil. The Espinhaço range is large and extends almost continuously from northeastern to southeastern Brazil (over 1,200 km) [52], has unique characteristics and geographically divides three of the main Brazilian biomes—*Cerrado*, Atlantic forest, and *Caatinga*–which together host a diverse array of endemic plant and animal species [53,54]. The Caraça Mountains host a protected area composed of heterogeneous habitat with different phytophysiognomies [55]. This area contains the highest peaks and has the greatest variation in altitude across the Espinhaço range (between 850 and 2,072 m.a.s.l.), where *campos rupestres* define a significant portion of the vegetation structure [56]. *Campos rupestres* are rocky mountaintop, neotropical, azonal vegetation complexes [57]. This region contains old, climate-buffered, and infertile landscapes (OCBILs), with probably the most ancient open vegetation in eastern South America [54].

### Sampling design

**Fig 1:**
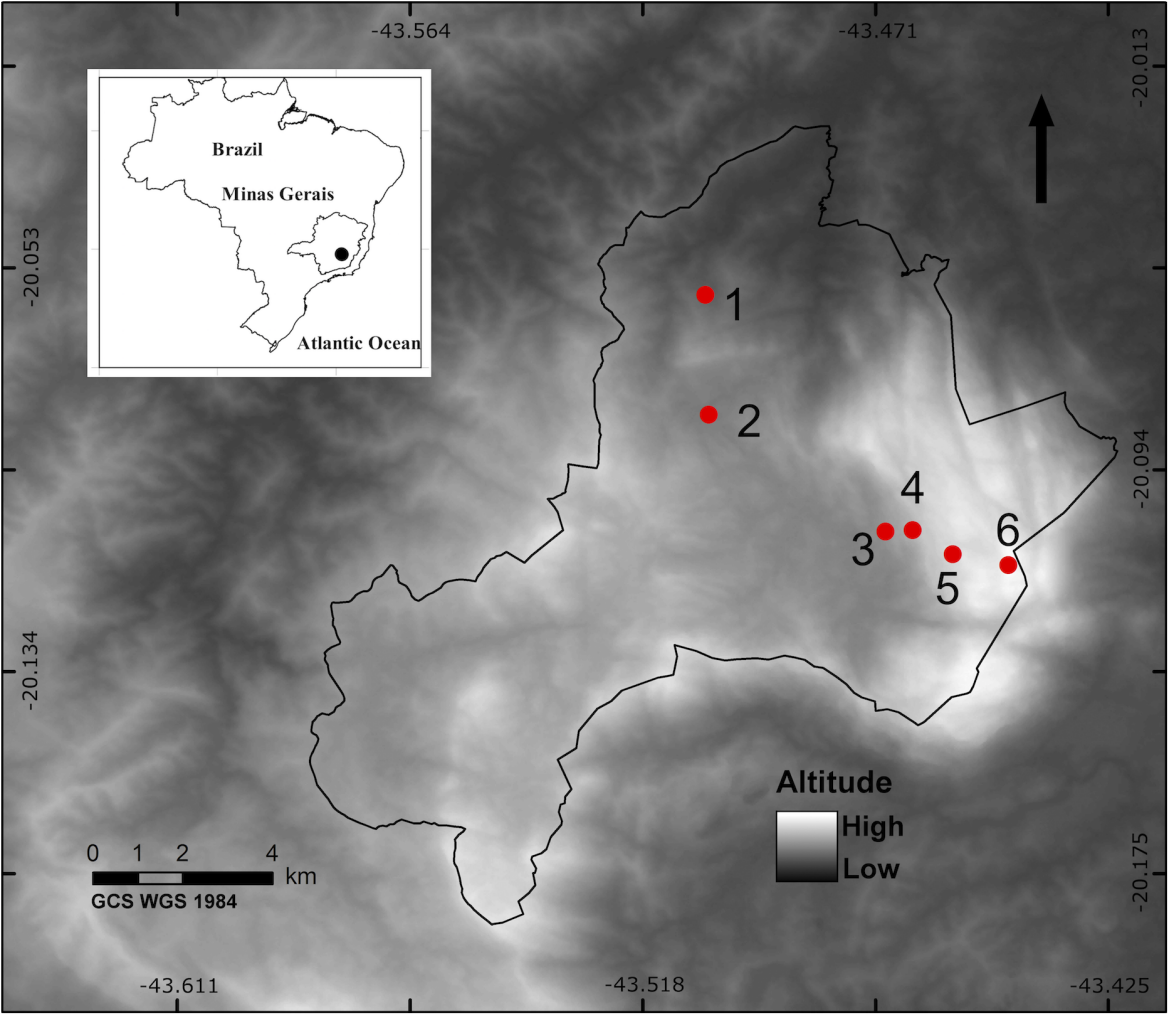
Location and distribution of sampling plots at RPPN *Santuário do Caraça*, Minas Gerais, Brazil. Numbers represent the six sampling plots. Plot altitude was as follows: 1 = 1,000; 2 = 1,200; 3 = 1,400; 4 = 1,600; 5 = 1,800; 6 = 2,000 m.a.s.l.

**Fig 2:**
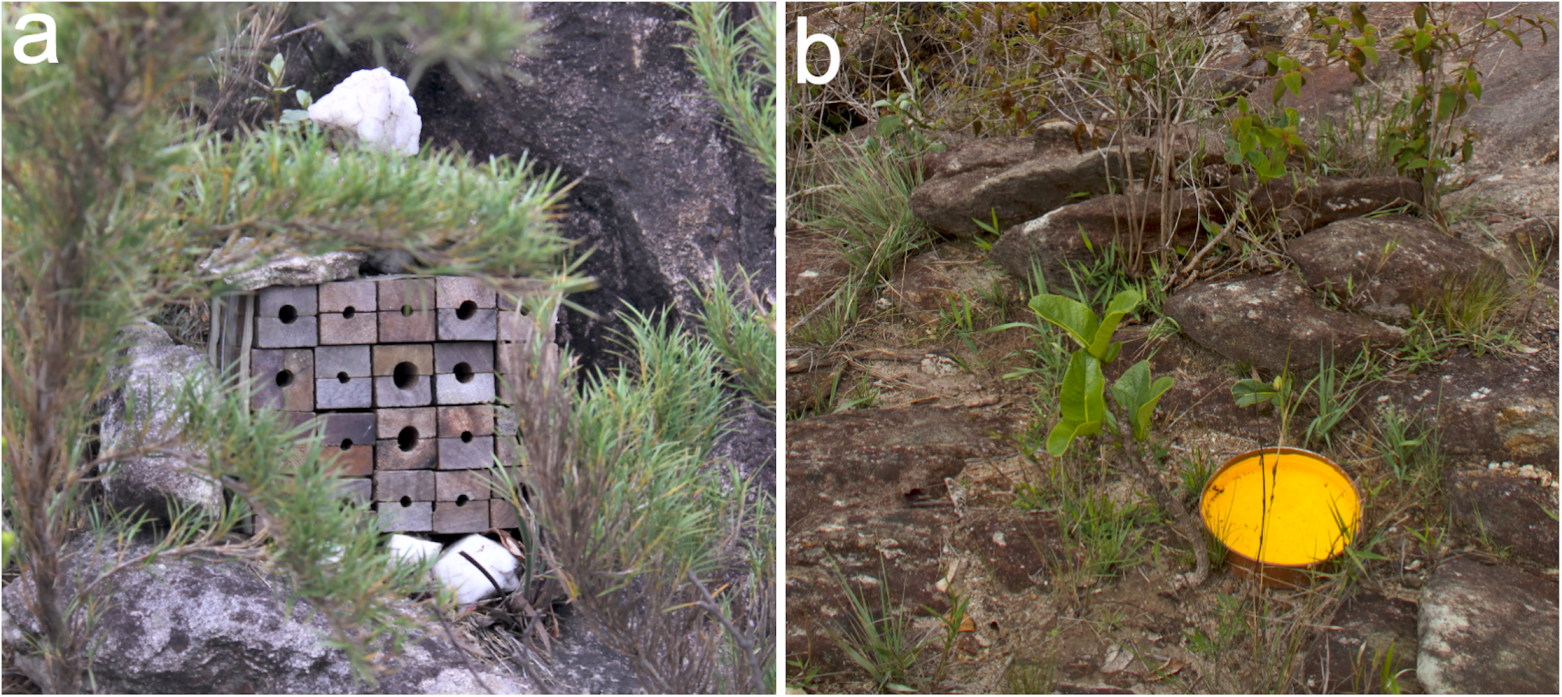
**Trap nests (a) and Moericke traps (b).** Nine trap nest packs containing twenty nests each, and nine Moericke traps were placed at each sampling plot.

We collected Aculeate wasps and bees in the Caraça Mountains at six sampling plots with different elevations, with altitudes between 1,000 and 2,000 m.a.s.l. (under ICMBio permission 20493–1). There was a 200 m difference in altitude between plots (Fig 1). We arranged packs containing twenty trap nests, consisting of 25 x 25 x 130 mm wood pieces with a central hole (11 cm depth) and diameters of 6, 9 and 12 mm (Fig 2A). Nine packs were placed at each plot with a distance of 50 m between packs, totaling 180 trap nests per sampling plot. Trap nests were inspected every two weeks for 12 months (17,820 trap-days). Traps colonized by Aculeata species were collected and replaced, then taken to the laboratory for monitoring until adult emergence. We also set nine Moericke traps at each plot (yellow, 25 cm diam. container filled with salty liquid, N = 54) (Fig 2B), which were placed directly on the ground with a distance of 50 m between traps. Moericke traps were placed in the field during trap nest monitoring, where they remained for 48 hours per sampling period. There was a minimum of 15 days between sampling, which over 12 months resulted in 18 sampling events (46,656 trap-hours).

All captured individuals belonging to Aculeate hymenopteran families were used in the study, with the exception of Formicidae (because our methodology was inappropriate for sampling these species) and Bethylidae (due to identification issues). Bees and wasps were identified to the lowest taxonomic level possible, based on Fernández & Sharkey keys [58] and also consulted specialists. Specimens were deposited in the *Coleção Entomológica da Universidade Federal de Minas Gerais* (CEUFMG) in Belo Horizonte, Minas Gerais, Brazil.

We obtained mean annual temperature (°C), precipitation (mm), solar radiation (kJ m^-2^ day^-1^), wind speed (m s^-1^) and water vapor pressure (kPa) at each site using Wordclim version 2 (1970–2000). We then tested for effects of these environmental variables on species richness and composition at each altitude.

### Statistical analysis

Generalized linear models (GLMs) were used to determine the influence of altitude on Aculeata species richness and abundance. The residuals from the GLMs were assessed to determine the appropriateness of the error distribution [59] using the ‘rdiagnostic’ procedure in the RT4Bio package, poisson and negative binomial regression models were used. We first ran correlation analyses to explore relationships between altitude and explanatory variables from Worldclim v2, and explanatory variables with high correlation values—Pearson’s correlation coefficient (r) greater than 0.7—were grouped into single variables for further analyses (S1 Fig) (see [60,61]). Non-correlated variables were included in GLM, and model simplification methods were used to identify relative importance. Sampling efficiency was analyzed using total species accumulation curves. A non-parametric estimator (Jackknife 1) was used to estimate total species richness for all sampling events at all altitudes.

We used a Mantel test to evaluate the distance decay of similarity (see Nekola and White 1999) among sample plot altitudes. We analyzed association patterns between distance matrices [62] using Jaccard dissimilarities for Aculeata species composition, and Euclidean distance matrices for altitudinal distance with 10,000 permutations.

To test the relative contributions of the two components of beta-diversity (species turnover and nestedness) across altitudes, we decomposed total β-diversity (represented by Sorensen dissimilarities: β_SØR_) for multiple sites [13,63,64]. This approach allows us to calculate the relative contribution of each component—species replacement (Simpson dissimilarity: β_SIM_) and nestedness (β_SØR_ - β_SIM_ = β_SNE_) to total β-diversity [13] (in percentage).

We used R v.3.3.1 [65] to perform all statistical analyses. We used the ‘Psych’ package to calculate the correlation coefficient among WorldClim variables. Mantel tests and calculations of Euclidean distances between altitude pairs were carried out using ‘vegan’ and ‘ecodist’ packages, and the ‘betapart v.1.3’ package was used to partition beta diversity into turnover and nestedness components (see [66]).

## Results

### Community analyses

**Fig 3:**
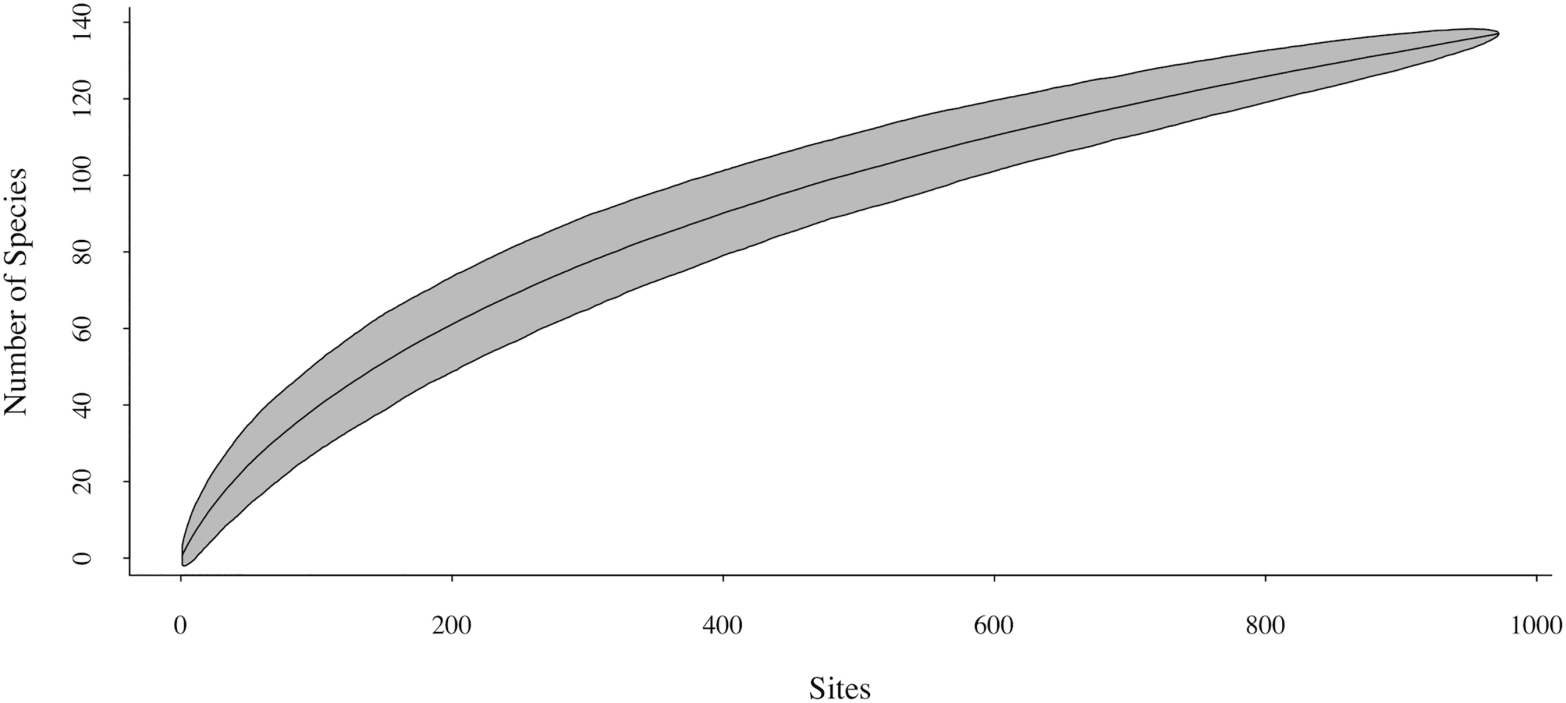
Species accumulation curve for Aculeate wasps and bees. Analyses using Jackknife 1 estimates. Specimens were collected at the RPPN Santuário do Caraça, Minas Gerais, Brazil.

We collected 1,306 specimens distributed among 137 morphospecies and 14 families (S1 Table). The species accumulation curve did not reach an asymptote (Fig 3). According to the Jackknife 1 estimator, sampling adequacy was 69.92% (observed richness: 137; estimated richness: 195.94). We found a high number of rare species, with 58 singletons (42.34% of the total) and 17 doubletons (12.41% of the total).

Moericke traps captured 124 morphospecies belonging to 13 families. In 63 occupied trap nests (5.5% of the total), we captured 17 wasp and bee species belonging to six families (Table 1). Only four species—*Caenochrysis* sp.1 (Chrysididae), *Trypoxylon* sp.1, *Trypoxylon* sp.7 (Crabronidae), and *Penepodium* sp. (Sphecidae)–were captured using both collection methods. The Eumeninae *Stenonartonia mimica* (Kohl 1907) (Vespidae) was captured in a trap nest located at 1,000 m.a.s.l., representing the first such observation for the state of Minas Gerais as well as the northernmost latitude described for this species (20°03'31.8'' S—43°30'19.7'' W) [67].

**Table 1:** List of Aculeata morphospecies found occupying trap nests at different altitudes in the Caraça Mountains, Brazil.

Morphospecies	Number of occupied nests	Emergence (Total)	Emergence per altitudes					
			1	2	3	4	5	6
**VESPOIDEA**								
**Vespidae**								
**Eumeninae**								
*Monobia angulosa*	9	30		30				
*Ancistroceroides* sp.	1	2			2			
*Stenonartonia mimica*	1	2	2					
**APOIDEA**								
**Sphecidae** **Sphecinae**								
*Penepodium* sp.	2	3			3			
**Crabronidae** **Crabroninae**								
*Trypoxylon lactitarse*	5	10		10				
*Trypoxylon* sp. 1	7	20		7		8	5	
*Trypoxylon* sp. 7	7	14	13	1				
**Apidae** **Apinae**								
*Centris (Heterocentris)*sp.	1	3	3					
*Centris (Hemisiella) tarsata*	14	53		45	8			
*Mesocheira bicolor*	1	5		5				
*Tetrapedia* sp.	1	1		1				
**Megachilidae** **Megachilinae**								
*Megachile (Dactylomegachile)* sp.	1	1				1		
*Megachile (Austromegachile)* sp.	4	13	13					
*Megachile (Moureapis)* sp.	1	6	6					
*Megachile (M*.*) anthidioides*	5	20		18	2			
**CHRYSIDOIDEA**								
**Chrysididae** **Chrysidinae**								
*Caenochrysis* sp.	2	3	1	2				
*Ipsiura* sp.	1	2	2					

Numbers represent the six sampling plots. Plot altitude was as follows: 1 = 1,000; 2 = 1,200; 3 = 1,400; 4 = 1,600; 5 = 1,800; 6 = 2,000 m.a.s.l.

All WorldClim variables were correlated with altitude except for solar radiation (S1 Fig), which had little importance in the GLM after model reduction. Therefore, altitude was used as the main explanatory variable for further analyses.

### Influence of altitude on species richness and abundance

**Fig 4:**
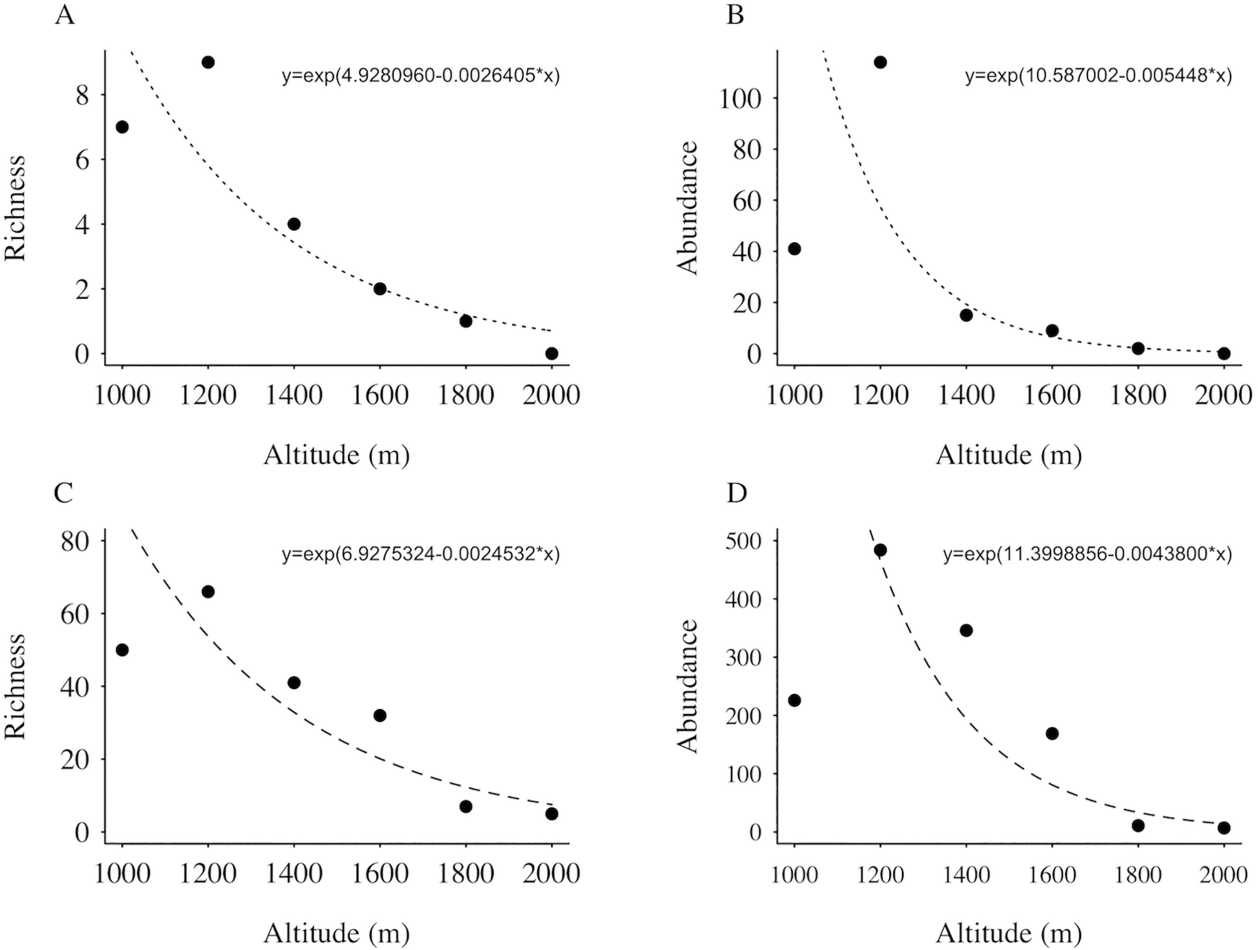
Relation of species richness and abundance with altitude. Species richness (a) and abundance (b) in trap nests, and species richness (c) and abundance (d) in Moericke traps along an altitudinal gradient in the Caraça Mountains, Minas Gerais, Brazil.

Aculeate hymenopteran richness and abundance were negatively correlated with altitude regardless of trap method used (*P* <0.001 for all tests) (Fig 4). At higher altitudes, fewer trap nests were occupied (four nests at 1,600 m, two at 1,800 m and none at 2,000 m; Fig 4A and Fig 4B and Table 1). The same pattern was found for Moericke traps (Fig 4C and Fig 4D).

**Fig 5:**
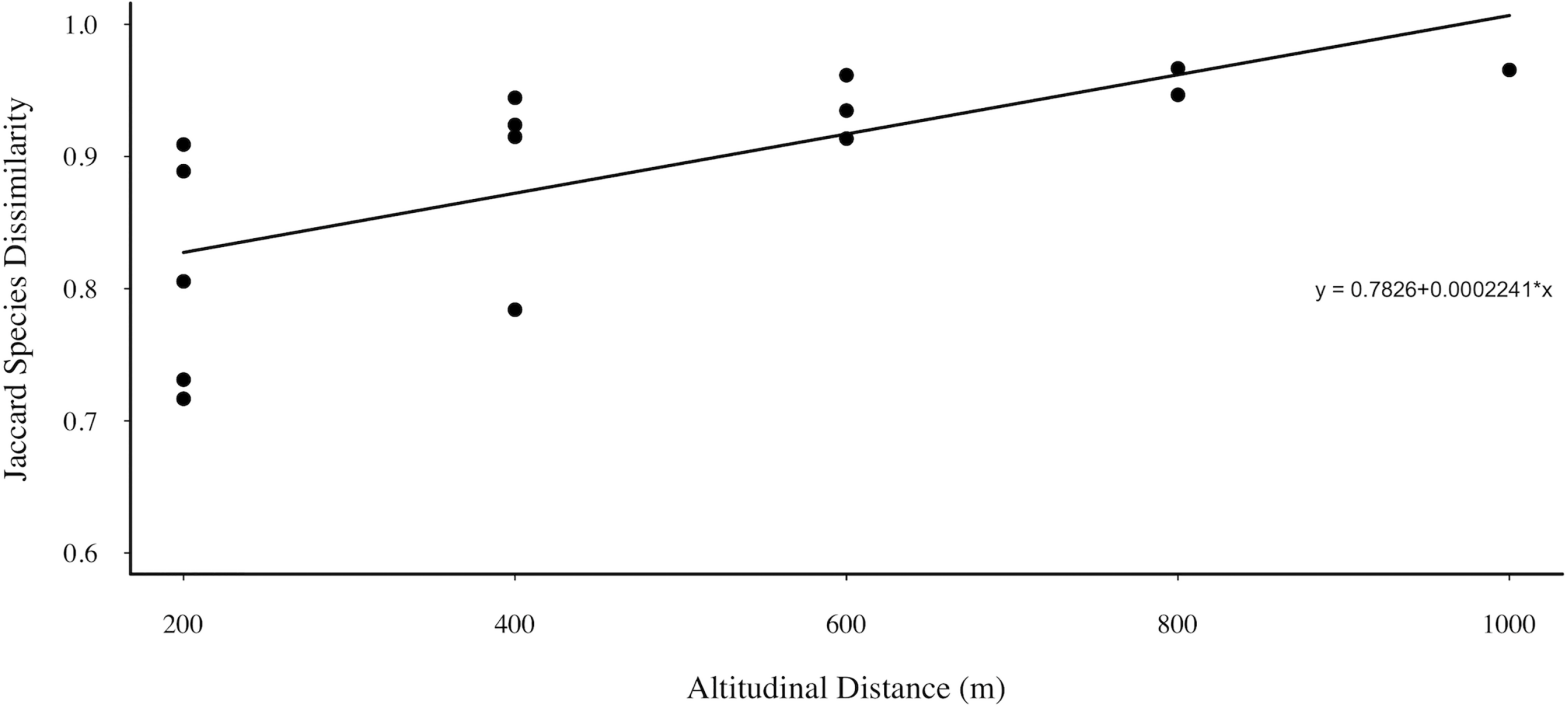
Relation of dissimilarity community composition with altitude. Schematic showing increasing Jaccard dissimilarity in Aculeate wasp and bee community composition with geographical altitudinal range (Mantel r = 0.85, *P* < 0.01).

Analysis of β-diversity patterns revealed a significant positive correlation between Aculeata pairwise community dissimilarity (β_SOR_) and the Euclidean distance of altitude among sites (Mantel r = 0.85, *P* < 0.01, Fig 5). β-diversity partitioning showed that effects were mainly due to species turnover, which accounted for 81% of total variation (compared to 19% for nestedness). The only species collected at all altitudes was *Trypoxylon* sp. 3 (Crabronidae).

## Discussion

As expected, bee and wasp richness and abundance in the Caraça Mountains decreased with increasing altitude, and relevant factors behind this explanation were correlated with altitude. Further, changes in species composition (i.e., β-diversity) occurred along the altitudinal gradient and increased with altitude range. β-diversity was primarily determined by species turnover as opposed to nestedness.

Similar negative correlations between diversity and altitude were found for other insect groups (see [17]), but the possible mechanisms that produce such patterns have been less explored. Hodkinson [68] indicated that along an elevational gradient, insect species experience significant differences in environmental conditions, and interactions with other organisms may also differ. However, Hodkinson claims that the mechanisms acting on altitudinal variation in species richness are still poorly understood. Possible mechanisms promoting these patterns have been considered in studies, including lower air humidity and temperature, and higher precipitation and wind intensity with increasing altitude [23,69]. These variables are known to reduce insect flight capability and activity [24,70]. These factors are present in mountain ecosystem, and are directly correlated with altitude especially temperature that is considered the main predictor of species richness in elevational biodiversity gradients [22]. Species richness and abundance were lower at 1,000 m than at 1,200 m. This may be explained by the fact that the lower altitude plot (1,000 m) had denser vegetation with greater numbers of trees. Areas with abundant and diverse tree communities typically have higher availability of natural cavities [71,72], and this may decrease occupancy rates for trap nests [73,74]. Some studies also show that yellow Moericke traps work better in open habitats, because traps must be visible in order to capture high numbers of insects [75,76].

Few studies have evaluated the influence of altitudinal gradients on hymenopterans in Brazilian montane ecosystems (including *campos rupestres*). Our results generally agree with those of Santos and Brandão [77] in their investigation of solitary Vespidae along an altitudinal gradient at the *Parque Estadual da Serra do Mar* (São Paulo State, Brazil). Martins et al. [78] argue that some stingless bee species (Apidae) cannot persist at high altitudes, mainly due to strong winds and intense cold. Azevedo et al. [48] did a survey in Espinhaço mountain range and collected nearly 360 bee species, but emphasized the lack of information for accurate determination of their geographical distributions.

Wasp and bee communities showed significant distance decay of community similarity across the altitudinal range, with β-diversity among communities increasing with increasing distance along the altitudinal gradient. This suggests environmental filtering and dispersion limitations between low elevations and mountaintops. Geographical distance is well known to influence community dissimilarity [34,79–81], however, investigations of these effects across altitudinal distances are less common. Some examples include studies of birds [16], tropical trees [82], insect pollinators [83], and ants [84], and all of these studies showed a significant, but not always strong, relationship between species composition and altitude.

Although it was not possible to identify an altitudinal threshold that separates lowland species from those of higher elevations (as found in Silveira and Cure [46]), species composition did change with altitude. Variation in β-diversity among altitudes was primarily due to species turnover, which has also been found for other insect groups [14,15,84,85]. Our results suggest that variables which change with altitudinal gradients may serve as environmental filters [27], contributing to species turnover and thus, β-diversity [14].

We did not find any exclusive species in trap nests in higher altitudes, a result also found by Morris et al. [21] wherein numbers of insects nesting in pre-existing cavities decreased with elevation. Conversely, using Moericke traps, we found two species restricted to higher altitudes (above 1,800 m.a.s.l.): *Polybia bifasciata* Saussure, 1854 (Vespidae) and *Ceratina (Crewella)* sp.3 (Apidae). Studies indicate that the species at higher elevations typically have greater altitudinal ranges and smaller geographic distributions [42]. Trap nests were found to be relatively unsuitable for altitudinal surveys, mainly due to well-known method selectivity and to the small capture rates for Aculeata species at higher elevations. Yoon et al. [86] found a significant decreasing rate of occupied trap nests when the altitude exceeded 800 m. In our work, only 1.1% (6 out of 540; two species) of available nests above 1,600 m.a.s.l were occupied, a small percentage rate when compared with studies performed at lower tropical sites. Taki et al. [87] found 612 occupied nests by 12 species from 1,728 nests available. Another similar study has shown that in a single month 36% of the total amount of trap nests were colonized by 11 different species [88].

Nevertheless, species richness and abundance in colonized nests was high compared to other studies in highland regions (see [89,90]). In addition to abiotic conditions, food and nesting resource availability determines the occurrence of some species [37,90,91]. Soil characteristics may also be important to nesting success [38,92], because at higher altitudes, soils become more shallow [41] and compact [78], and clay and silt present become increasingly rare. These high altitude soils may hinder nest construction for Aculeata species, which utilize these types of raw material for nest construction (especially exposed nests built by wasps) [93]. Interestingly, though artificial cavities were offered in the form of trap-nests, cavity colonization was not effective. This suggests that the cavities themselves are not the only nest-limiting resource.

Environmental variables caused β-diversity to change along the mountain elevation gradient, and species turnover is influenced by high rate of singletons and doubletons. This pattern is commonly seen in tropical arthropod studies [81,94,95], even those yielding abundant specimens. Bee fauna of the *campos rupestres* in the Espinhaço Mountain Range show low abundance and high numbers of rare species [48]. This pattern has also been found in other high mountain grasslands [17]. Most surveys are carried out as part of the licensing processes for large developments, such as mining, and are thus extremely short-term, with rare exceptions [51]. Our results highlight the need for long-term studies in order to fully assess hymenopteran diversity, specifically in mountainous areas. Our results also indicate that conservation of Aculeata diversity in tropical mountain systems such as *campos rupestres* will strongly depend on preservation of environmental heterogeneity across altitudinal strata.

## Supporting information

S1 Fig Pearson’s correlation coefficient (*r*) between altitude and WorldClim v2 variables (1970–2000). A *r* value greater than 0.7 were the parameter to consider correlated variables. Mean values of temperature (°C), precipitation (mm), solar radiation (kJ m^-2^ day^-1^), wind speed (m s^-1^) and water vapor pressure (kPa).
(TIFF)

S1 Table Aculeata species occurrences and distributions along an altitudinal gradient in the Caraça Mountains, Minas Gerais, Brazil. (PDF)
