# Supplementary figures and images for: Compositional changes in bee and wasp communities along Neotropical mountain altitudinal gradient
Source: PLoS One. 2017 Jul 26;12(7):e0182054. doi: 10.1371/journal.pone.0182054 (PMC5528900; doi:10.1371/journal.pone.0182054)

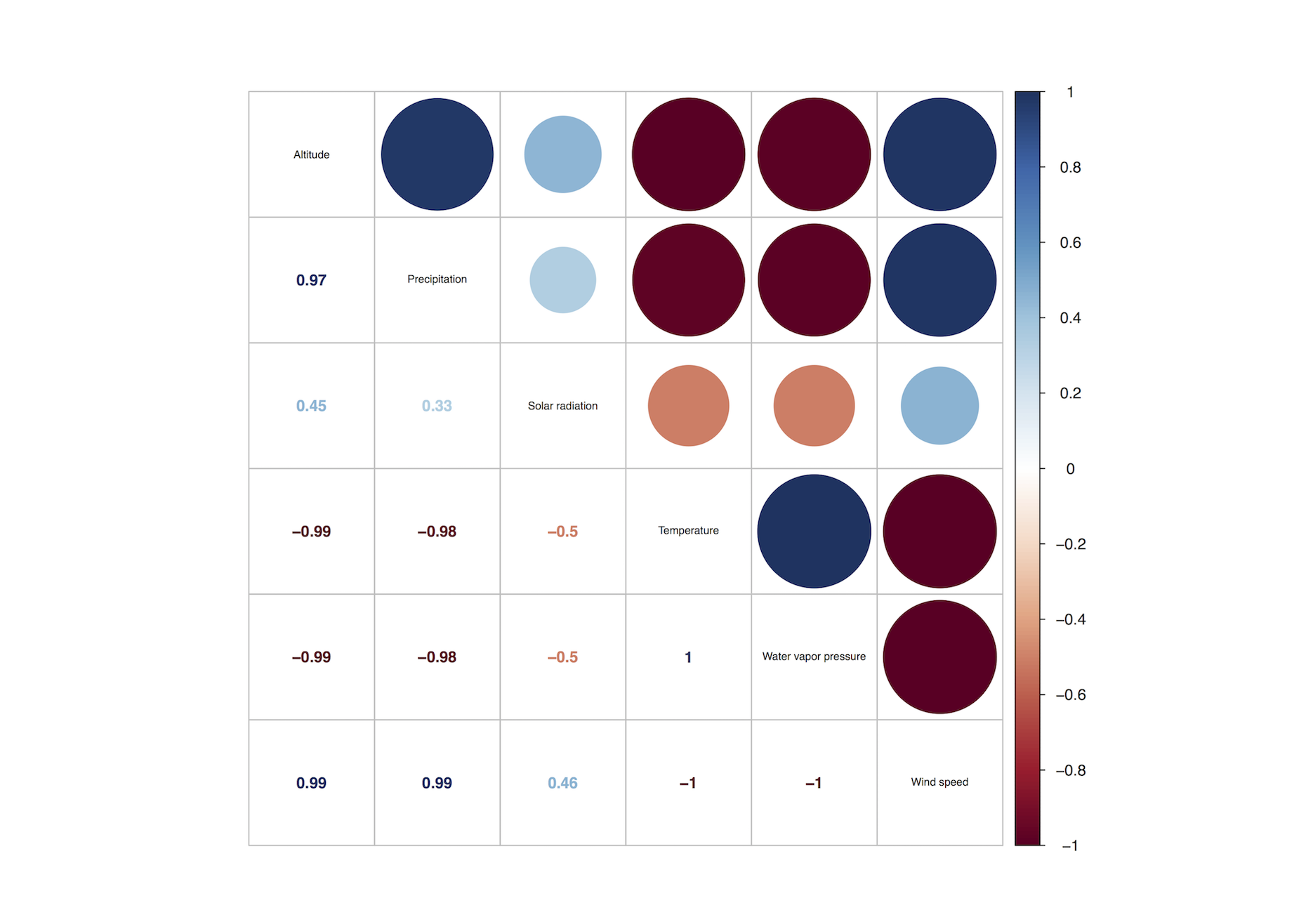

Supplement: S1 Fig — A r value greater than 0.7 were the parameter to consider correlated variables. Mean values of temperature (°C), precipitation (mm), solar radiation (kJ m-2 day-1), wind speed (m s-1) and water vapor pressure (kPa). (TIFF) [file pone.0182054.s001.TIFF]
