# Supplementary material for: Compositional changes in bee and wasp communities along Neotropical mountain altitudinal gradient
Source: PLoS One. 2017 Jul 26;12(7):e0182054. doi: 10.1371/journal.pone.0182054 (PMC5528900; doi:10.1371/journal.pone.0182054)
Supplement: S1 Table — (PDF) [file pone.0182054.s002.pdf]

S1 Table. Aculeata species occurrences and distributions along an altitudinal gradient in the Caraça Mountains, Minas Gerais, Brazil.

TÁXON	Capture method	Altitudes (m)						TOTAL
		1000	1200	1400	1600	1800	2000	
**VESPOIDEA**								
**Vespidae**								
**Polistinae**								
Polistes subsericeus Saussure, 1854	Moericke trap		4					4
Mischocyttarus drewseni (Saussure, 1857)	Moericke trap			1				1
Agelaia multipicta (Haliday, 1836)	Moericke trap	13	4					17
Agelaia myrmecophila (Ducke, 1905)	Moericke trap		6					6
Agelaia vicina (Saussure, 1854)	Moericke trap		23					23
Brachygastra cf. *fistulosa*	Moericke trap				1			1
Polybia bifasciata Saussure, 1854	Moericke trap					1		1
Polybia chrysothorax (Lichtenstein, 1796)	Moericke trap		1					1
Polybia dimidiata (Olivier, 1791)	Moericke trap		1					1
Polybia fastidiosuscula Saussure, 1854 Morph. *Buyssoni*	Moericke trap		4	1				5
Polybia fastidiosuscula Saussure, 1854 Morph. *fastidiosuscula*	Moericke trap			1			1	2
Polybia flavifrons Smith, 1857	Moericke trap			4		1		5
Polybia ignobilis (Haliday, 1836)	Moericke trap		1					1
Protopolybia sedula (Saussure, 1854)	Moericke trap		3					3
**Eumeninae**								
Ancistroceroides sp.	Trap nest			1				1
Stenodynerus sp.	Moericke trap		1					1
Monobia angulosa Saussure, 1852	Trap nest		9					9
Stenonartonia mimica (Kohl, 1907)	Trap nest	1						1
Omicron spegazzinii (Brèthes, 1905)	Moericke trap		1					1
Omicron tuberculatum (Fox, 1899)	Moericke trap		1	1				2
**Scoliidae**								
**Scoliinae**								
Campsomeris sp.1	Moericke trap		1					1
*Campsomeris*sp.2	Moericke trap			1				1
**Pompilidae**								
**Pompilinae**								
Agenioideus sp.	Moericke trap			1				1

TÁXON	Capture method	Altitudes (m)						TOTAL
		1000	1200	1400	1600	1800	2000	
*Allochares*cf. sp.	Moericke trap			1				1
Anoplius sp.1	Moericke trap	15	2					17
Anoplius sp.2	Moericke trap	1						1
Anoplius sp.3	Moericke trap		1		2			3
Aplochares sp.	Moericke trap			1				1
Aporinellus sp.	Moericke trap	3						3
Paracyphononyx sp.	Moericke trap		1					1
Poecilopompilus sp.	Moericke trap		1					1
Aporus sp.	Moericke trap				3			3
Psorthaspis sp.1	Moericke trap	4						4
Psorthaspis sp.2	Moericke trap		4	2	2			8
**Ceropalinae**								
Irenangelus sp.	Moericke trap	1						1
Ceropales cf. sp.	Moericke trap		1					1
**Cnetocerinae**								
Epipompilus cf. sp.1	Moericke trap	1	1	1				3
Epipompilus cf. sp.2	Moericke trap	2	1					3
**Pepsinae**								
Pepsinae sp.1	Moericke trap	1						1
Pepsinae sp.2	Moericke trap	1	1					2
Pepsinae sp.3	Moericke trap	6						6
Pepsinae sp.4	Moericke trap	3						3
Pepsinae sp.5	Moericke trap	1						1
Pepsinae sp.6	Moericke trap	1						1
Pepsinae sp.7	Moericke trap	4	1					5
Pepsinae sp.8	Moericke trap	2						2
Pepsinae sp.9	Moericke trap	1						1
Pepsinae sp.10	Moericke trap	4	1					5
Pepsinae sp.11	Moericke trap	9	3					12
Pepsinae sp.12	Moericke trap	6	6					12
Pepsinae sp.13	Moericke trap	1	2					3
Pepsinae sp.14	Moericke trap	1	3					4
Pepsinae sp.15	Moericke trap	4						4

TÁXON	Capture method	Altitudes (m)						TOTAL
		1000	1200	1400	1600	1800	2000	
Pepsinae sp.16	Moericke trap	2			3			5
Pepsinae sp.17	Moericke trap	1						1
**Mutilidae**								
**Mutilinae**								
Ephuta sp.	Moericke trap	2						2
Timulla sp.1	Moericke trap		1		1			2
Timulla sp.2	Moericke trap				1			1
**Sphaerophtalminae**								
Sphaeropthalmina sp.	Moericke trap	1						1
Pseudomethocina sp.1	Moericke trap	4			1			5
Pseudomethocina sp.2	Moericke trap	1						1
Xystromutilla cf. sp.	Moericke trap	1						1
**Tiphiidae**								
Tiphiidae sp.1	Moericke trap		1					1
Tiphiidae sp.2	Moericke trap		5		1			6
Tiphiidae sp.3	Moericke trap	5	15		2			22
Tiphiidae sp.4	Moericke trap		5	5	11			21
Tiphiidae sp.5	Moericke trap		1					1
Tiphiidae sp.6	Moericke trap	4						4
Tiphiidae sp.7	Moericke trap	1						1
**APOIDEA**								
**Sphecidae**								
**Sphecinae**								
Penepodium sp.	Moericke trap/Trap nest			1/2				1/2
Podium cf. sp.	Moericke trap			2				2
Ammophila sp.	Moericke trap		1					1
**Ampulicidae**								
**Ampulicinae**								
Dolichurus sp.	Moericke trap		25	15	37			77
Paradolichurus sp.	Moericke trap		2					2
**Crabronidae**								
**Crabroninae**								
Larra sp.1	Moericke trap		1	1				2

TÁXON	Capture method	Altitudes (m)						TOTAL
		1000	1200	1400	1600	1800	2000	
Larra sp.2	Moericke trap		1					1
Liris sp.1	Moericke trap	26	63	128	17			234
Liris sp.2	Moericke trap	3	25	7				35
*Tachysphex*sp.1	Moericke trap		25	17	28			70
*Tachysphex*sp.2	Moericke trap		3	3	2			8
*Tachysphex*sp.3	Moericke trap		1					1
Trypoxylon lactitarse Saussure, 1867	Trap nest		5					5
Trypoxylon sp.1	Moericke trap/Trap nest	1/0	3/2	39/0	23/3	5/2		71/7
Trypoxylon sp.2	Moericke trap	1						1
Trypoxylon sp.3	Moericke trap	9	2	5	2	1	1	20
Trypoxylon sp.4	Moericke trap		4	1	1			6
Trypoxylon sp.5	Moericke trap		1	3				4
Trypoxylon sp.6	Moericke trap	4						4
Trypoxylon sp.7	Moericke trap/Trap nest	1/6	0/1					8
Pison (pison) sp.1	Moericke trap		3	5	4			12
Pison (pison) sp.2	Moericke trap		2					2
*Nitela*sp	Moericke trap		6	1				7
Bembicinae								
Nyssonini sp.1	Moericke trap		1					1
Nyssonini sp.2	Moericke trap				1			1
Nyssonini sp.3	Moericke trap		1					1
Nyssonini sp.4	Moericke trap		2		4			6
**Apidae**								
**Apinae**								
Apis mellifera (Linnaeus, 1758)	Moericke trap		8	5	4	1		18
*Centris (Hemisiella) tarsata*Smith, 1874	Trap nest		12	2				14
Centris (Heterocentris) sp.	Trap nest	1						1
Mesocheira bicolor (Fabricius, 1804)	Trap nest		1					1
Trigona guianae Cockerell, 1910	Moericke trap	11	1					12
Trigona spinipes (Fabricius, 1793)	Moericke trap	50	180	32	4		1	267
Geotrigona sp.	Moericke trap		2	47	2			51
Melipona (Eomelipona) bicolor Lepeletier, 1836	Moericke trap	3	1					4
Melipona (melipona) quadrifasciata Moure 1992	Moericke trap	1						1

TÁXON	Capture method	Altitudes (m)						TOTAL
		1000	1200	1400	1600	1800	2000	
Paratrigona subnuda Moure, 1947	Moericke trap			1				1
Partamona ailyae Camargo, 1980	Moericke trap			1				1
Partamona cf. *combinata* Pedro & Camargo, 2003	Moericke trap	3						3
Partamona cf. *nigrilabris* Pedro & Camargo, 2003	Moericke trap	1						1
Partamona sp.	Moericke trap	1		1				2
Paratetrapedia (Xanthopedia) sp.	Moericke trap			1				1
**Xylocopinae**								
Tetrapedia sp	Trap nest		1					1
Ceratina (Ceratinula) sp.	Moericke trap		1				1	2
Ceratina (Crewella) sp.1	Moericke trap				1			1
Ceratina (Crewella) sp.2	Moericke trap				1			1
Ceratina (Crewella) sp.3	Moericke trap					1		1
Ceratina (Crewella) sp.4	Moericke trap				1	1		2
**Andrenidae**								
**Panurginae**								
*Anthrenoides*sp.1	Moericke trap		1	3	2			6
Anthrenoides sp.2	Moericke trap			1	1			2
Anthrenoides sp.3	Moericke trap			1	1			2
Anthrenoides sp.4	Moericke trap			1				1
**Halictidae**								
**Halictinae**								
Dialictus sp.1	Moericke trap			1	2			3
Dialictus sp.2	Moericke trap		2				3	5
Dialictus sp.3	Moericke trap				3			3
Dialictus sp.4	Moericke trap		5	1				6
Augochlora (*Augochloras.str*.) sp.1	Moericke trap		1					1
Augochloropsis sp.1	Moericke trap		1	1				2
Augochloropsis sp.2	Moericke trap			1				1
Augochloropsis sp.3	Moericke trap		1					1
**Megachilidae**								
**Megachilinae**								
Megachile (Austromegachile) sp.	Trap nest	4						4
Megachile (Dactylomegachile) sp.	Trap nest				1			1

TÁXON	Capture method	Altitudes (m)						TOTAL
		1000	1200	1400	1600	1800	2000	
Megachile (Moureapis) anthidioides Radoszkowski, 1874	Trap nest		4	1				5
Megachile (Moureapis) sp.	Trap nest	1						1
**Colletidae**								
**Paracolletinae**								
Niltonia sp.	Moericke trap	1						1
**CHRYSIDOIDEA**								
**Chrysididae**								
**Chrysidinae**								
Caenochrysis sp.1	Moericke trap/Trap nest	1/1	0/1					1/2
Caenochrysis sp.2	Moericke trap	2						2
Ipsiura sp.	Trap nest	1						1
